# PHB/CHIT Scaffold as a Promising Biopolymer in the Treatment of Osteochondral Defects—An Experimental Animal Study

**DOI:** 10.3390/polym13081232

**Published:** 2021-04-11

**Authors:** Eva Petrovova, Marek Tomco, Katarina Holovska, Jan Danko, Lenka Kresakova, Katarina Vdoviakova, Veronika Simaiova, Filip Kolvek, Petra Hornakova, Teodor Toth, Jozef Zivcak, Peter Gal, David Sedmera, Lenka Luptakova, Lubomir Medvecky

**Affiliations:** 1 Department of Morphological Disciplines, University of Veterinary Medicine and Pharmacy in Kosice, Komenskeho 73, 04181 Kosice, Slovakia; katarina.holovska@uvlf.sk (K.H.); jan.danko@uvlf.sk (J.D.); lenka.kresakova@uvlf.sk (L.K.); katarina.vdoviakova@uvlf.sk (K.V.); veronika.simaiova@uvlf.sk (V.S.); lmedvecky@saske.sk (L.M.); 2 Railway Hospital in Kosice, Masarykova 1632/9, 04001 Kosice, Slovakia; mtomco.75@gmail.com; 3 Clinic of Horses, University of Veterinary Medicine and Pharmacy in Kosice, Komenskeho 73, 04181 Kosice, Slovakia; filip.kolvek@student.uvlf.sk (F.K.); petra.hornakova@uvlf.sk (P.H.); 4 Department of Biomedical Engineering and Measurement, Faculty of Mechanical Engineering, Technical University of Kosice, Letna 9, 04200 Kosice, Slovakia; teodor.toth@tuke.sk (T.T.); jozef.zivcak@tuke.sk (J.Z.); 5 Center of Clinical and Preclinical Research MediPark, Pavol Jozef Safarik University, 04011 Kosice, Slovakia; peter.gal@upjs.sk; 6 Department of Biomedical Research, East-Slovak Institute of Cardiovascular Diseases, Inc., 04011 Kosice, Slovakia; 7 Prague Burn Centre, Third Faculty of Medicine, Charles University and University Hospital Kralovske Vinohrady, 10034 Prague, Czech Republic; 8 Institute of Anatomy, Charles University, U nemocnice 3, 12800 Prague, Czech Republic; david.sedmera@lf1.cuni.cz; 9 Institute of Physiology, The Czech Academy of Sciences, Videnska 1083, 14200 Prague, Czech Republic; 10 Department of Biology and Physiology, University of Veterinary Medicine and Pharmacy in Kosice, Komenskeho 73, 04181 Kosice, Slovakia; lenka.luptakova@uvlf.sk; 11 Institute of Materials Research, The Slovak Academy of Sciences, Watsonova 1935/47, 04001 Kosice, Slovakia

**Keywords:** biopolymer, cartilage, chitosan, regeneration, sheep model

## Abstract

Biopolymer composites allow the creation of an optimal environment for the regeneration of chondral and osteochondral defects of articular cartilage, where natural regeneration potential is limited. In this experimental study, we used the sheep animal model for the creation of knee cartilage defects. In the medial part of the trochlea and on the medial condyle of the femur, we created artificial defects (6 × 3 mm^2^) with microfractures. In four experimental sheep, both defects were subsequently filled with the porous acellular polyhydroxybutyrate/chitosan (PHB/CHIT)-based implant. Two sheep had untreated defects. We evaluated the quality of the newly formed tissue in the femoral trochlea defect site using imaging (X-ray, Computer Tomography (CT), Magnetic Resonance Imaging (MRI)), macroscopic, and histological methods. Macroscopically, the surface of the treated regenerate corresponded to the niveau of the surrounding cartilage. X-ray examination 6 months after the implantation confirmed the restoration of the contour in the subchondral calcified layer and the advanced rate of bone tissue integration. The CT scan revealed a low regenerative potential in the bone zone of the defect compared to the cartilage zone. The percentage change in cartilage density at the defect site was not significantly different to the reference area (0.06–6.4%). MRI examination revealed that the healing osteochondral defect was comparable to the intact cartilage signal on the surface of the defect. Hyaline-like cartilage was observed in most of the treated animals, except for one, where the defect was repaired with fibrocartilage. Thus, the acellular, chitosan-based biomaterial is a promising biopolymer composite for the treatment of chondral and osteochondral defects of traumatic character. It has potential for further clinical testing in the orthopedic field, primarily with the combination of supporting factors.

## 1. Introduction

Hyaline cartilage or articular cartilage (AC) is the most common form of cartilage and it covers the articular surfaces in synovial joints. This specialized tissue provides a smooth, cushioning, and low-friction surface for joints [1]. Avascular nature and limited ability to self-repair of AC are challenging factors for successful tissue regeneration. Mechanical damage of AC either by an injury or by breakdown due to ageing may lead to irreversible consequences directly affecting quality of life [2]. 

Even injuries of the AC lead to progressive damage and osteoarthritic joint degeneration, which is one of the global leading causes of significant pain, disability, and immobility [3,4,5,6]. Osteoarthritis affects around 3.3–3.6% of the global population; it is the eleventh most debilitating disease causing moderate to severe disability in 43 million people [7,8]. Most clinical and research efforts currently focus on the restoration of damaged cartilage; however, success remains limited [9]. The first problem is to fill the defect void with tissue that has the same mechanical properties as the articular cartilage. The second is to promote successful integration between the repair tissue and the native articular cartilage [10].

Several methods have been developed by researchers and surgeons in attempts to regenerate or repair AC. The goal is to create and restore the function of hyaline cartilage; unfortunately, the result has many times shown to be newly formed fibrocartilage instead. That is a poor substitute for hyaline cartilage and the need for regeneration of more durable cartilage persists. The repair of cartilage can be improved by surgical intervention, but the current methods often lead to regeneration of this lower-quality fibrocartilage and the repair is only temporary. Animal models in cartilage repair studies have been of major importance for the development of effective treatments of cartilage injuries. For the use of new therapies in clinical practice, in vivo animal studies are necessary as a gap between in vitro experiments and human clinical studies [11]. In recent decades, large animal models, such as small ruminants (sheep and goats), pigs, dogs, and horses have been used to study the physiopathology and to develop new therapeutic procedures to treat human osteoarthritis. For that purpose, osteochondral defects are generally performed in the stifle joint of selected large animal models at the condylar and trochlear femoral areas where spontaneous regeneration should be excluded [12]. The ovine stifle joint defect model is widely accepted as an adequate model and is commonly used for the assessment of the efficacy of strategies for cartilage regeneration due to functional and morphological similarity between sheep cartilage and human cartilage in terms of structure, width, and density. Therefore, sheep are considered one of the most convenient animals for use in experimental studies of biopolymer-based (osteo)-chondral defect regeneration [13,14].

Regenerative medicine and tissue engineering provide us with promising alternatives to current clinical strategies based on the combination of biodegradable scaffolds and particular biological factors, such as growth factors or genetic cues [15]. Some natural materials that have been explored as bioactive scaffolds for cartilage engineering include chitosan, collagen, fibrin, alginate, and agarose [14,16]. Chitosan-based materials have been recognized as a suitable microenvironment for chondrocyte adhesion, proliferation, and differentiation when forming articular cartilage [17]. Chitosan has many attractive properties including biocompatibility, biodegradability, non-toxicity, remarkable affinity to proteins, bacterial resistance, and hemostaticity [18]. It is available in different forms (films, gels, sponges, fibers, nanoparticles, nanofibers), and thus is suitable for diverse applications in tissue engineering [19], promoting the healing process of soft and hard connective tissues [20]. Recently, in vitro studies have suggested that chitosan could promote cartilage-specific protein expression and reduce inflammatory and catabolic mediator production by chondrocytes [21]. Moreover, chitosan prevented cartilage degradation and synovial membrane inflammation in a rabbit model with induced OA [22,23]. The poly(3-hydroxybutyrate; PHB) represents natural or synthetic biodegradable and hydrophobic biopolymer, whereas the hydroxybutyric acid is a degradation product found in the body similar to glycolic acid and lactic acid as a normal metabolite [24]. Its biocompatibility and degradability make it a usable biomaterial for use in bone and cartilage regenerative medicine [25]. Accordingly, in vitro experiments conducted on fibroblasts and osteoblasts revealed no evidence of cytotoxicity of a porous PHB/chitosan biopolymer scaffold prepared by a modified method, and this material stimulated chondrogenesis at the in vitro level [26].

To evaluate the success and outcome of cartilage regeneration, one of the most important measures is the histological quality of cartilaginous tissue. Presently, a variety of histological scoring systems is used to evaluate and describe the quality of in vivo repaired tissue. Histological evaluation of repaired cartilage tissue is a proven and longstanding method for the assessment of quality with both qualitative and quantitative parameters [27]. O’Driscoll and the simple Pineda scales are reliable semi-quantitative cartilage scoring systems with good correlation. They are often used for the evaluation of the cartilage healing process in experimental animals [28,29]. These systems are among the most commonly used systems for experimental cartilage repair, and histological analysis is considered a gold-standard method for evaluation of cartilaginous repair tissue.

The aim of our study was to evaluate the healing process of osteochondral defects in the sheep stifle joint supported by acellular porous PHB/CHIT scaffold. Evaluation of the new repaired cartilage in the sheep animal model took place six months after the scaffold implantation using non-invasive imaging techniques (X-ray, CT, Magnetic Resonance Imaging (MRI)) and histological methods.

## 2. Materials and Methods

### 2.1. Preparation and Characterization of Composite Scaffold

**Figure 1:**
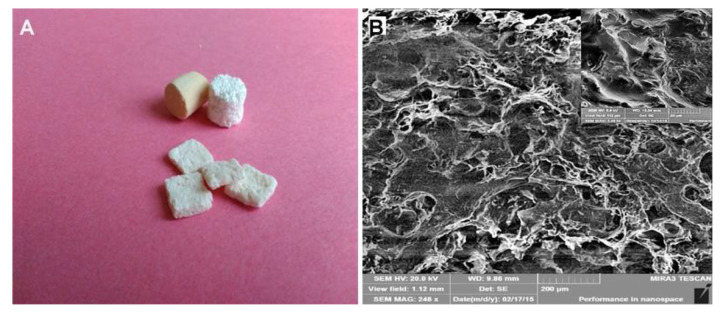
PHB/CHIT biomaterial. (**A**)—Macroscopic design of the used scaffold for the filling of the osteochondral defect; (**B**)—Microstructure of PHB/CHIT scaffold with pores (SEM), scale bar: 200 µm.

The PHB/CHIT biomaterial was prepared according to the previously published procedure [30]. PHB (GoodFellow, Cambridge, England) was diluted in propylene carbonate (1% sol. of PHB) and mixed in a 1/1 ratio with chitosan (Sigma–Aldrich, St. Louis, MO, USA, 1% sol. in 1% acetic acid) in a magnetic stirrer at a 400 rpm. After 10 min of stirring, acetone (about 5 mL) was added to the suspension to induce precipitation of the biopolymers and suspension was neutralized by NH_3_ (aq., 20%, Sigma–Aldrich, for High Performance Liquid Chromatography (HPLC)) after 5 min mixing. The final mixture was subsequently filtered and washed in distilled water and compressed into the final product of cylinders with a diameter of 6 mm and 10 mm thickness (Figure 1). The final products were then lyophilized (Ilshin, BX Ede, The Netherlands) for 6 h. Swelling of scaffolds was measured in 1.5 mL vials by immersion of porous substrates (approximately 20 mg) in saline solution at 37 °C up to a constant mass. Soaking was done in triplicate and swelling was evaluated as the ratio of weight of the wet sample to the original dry sample. The microstructure of scaffolds was observed by scanning electron microscopy (FE SEM JEOL7000). The phase analysis of blend was evaluated using the X-ray powder diffraction analysis (XRD, Philips X Pert Pro). The average molecular weights (Mw) of PHB and CHIT polymers in blends were determined by GPC [30] at level 80 and 28 kDa, respectively. The macroporous (around 85% porosity) and spongy-like microstructure with irregularly shaped macropores (sizes up to 100 µm) and wide distribution of micropores (<20 µm) was composed of the larger plate-like particles characteristic for chitosan and the fine microporous agglomerates with both the fibrous and more granular morphologies representing PHB.

The material is nontoxic, and no toxic solutions were used in its preparation and production [31]. Samples were sterilized in an autoclave at 121 °C (Gemmy SA-250MA) and no additional growth factors or cytokines were added prior to its use. Tested material was applied as an acellular structure without mesenchymal stem cells or autologous chondrocytes.

### 2.2. Animals

The study was carried out on six clinically healthy female sheep of the crossbreed of Merino and Valachian obtained from a farm PD Agro (Michalovce, Slovakia). The animals were of 1.5–2 years of age with an average body weight of 60 kg. They were housed in free stalls with free access to fresh water and food. The animals were included in the study 30 days before the scheduled day of surgical intervention, allowing adaptation to the changed environment. Before the inclusion into the study, the animals underwent standard preoperative clinical examination. It included the assessment of overall health status (food intake, behavior), inspection and recording of body temperature, respiratory and pulse rates, and a detailed evaluation of the organ systems [32]. After surgical intervention, the health status of the animals was evaluated daily until the end of the experimental study, and was oriented to the observation of the general health state after the surgical procedure, as well as local signs of inflammation.

In vivo experiments were performed according to European and Slovakian Law on animal models, after the approval of the research protocol by the Ethical Committee of University of Veterinary Medicine and Pharmacy in Kosice and responsible public authorities from the State Veterinary and Food Administration of the Slovak Republic (No. 2220/17-221) in agreement with EU regulations (EU Directive 2010/63/EU for animal experiments).

### 2.3. Surgical Manipulation with Biopolymer Composites

**Figure 2:**
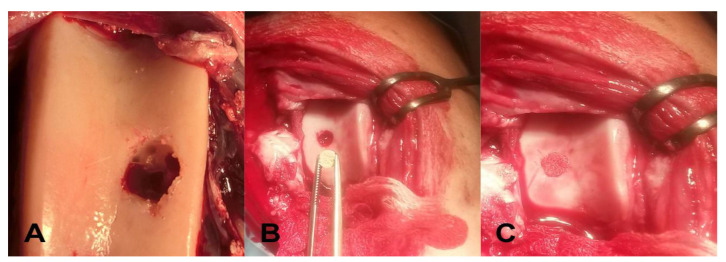
Surgical procedure. (**A**)—Formation of the artificial defect; (**B**)—Biomaterial prepared for implantation; (**C**)—Implanted biomaterial.

Surgical procedures were performed on the sheep under general anesthesia (buthorphanol 0.1 mg/kg i.m., Butomidor 10 mg/mL, Richter Pharma, Wels, Austria; medetomidin 0.02 mg/kg i.m., Cepetor 1mg/mL, CP-Pharma Handelsgesellschaft, mbH, Burgdorf, Germany, ketamine 8 mg/kg i.v., Ketamidor 100 mg/mL, Richter Pharma, Wels, Austria) and all basic vital functions were monitored during the surgery. After the shaving of the surgical region on the left knee, access was made from the lateral side. The incision extended from the medial patellar ligament distal to the tibial tuberosity. The weight-bearing area of the medial femoral condyle was exposed, and the subcutaneous tissue and superficial fascia were also dissected. After the flexion of the dissected knee and the dislocation of the patella, an artificial chondral lesion on the medial femoral condyle and the trochlear groove was performed using a negative cutter, a surgical curette, and an oblique chisel in a standard diameter of 6 mm and a depth of 3 mm. Three or four microdrills were performed at the base of each defect using a 0.33 mm Kirschner wire to form a communication with the cancellous bone layer as a source of multipotent mesenchymal stem cells. Both defects in 4 experimental animals were subsequently filled with a PHB/CHIT scaffold (diameter of 6 mm and depth of 3 mm; Figure 2). Two remaining animals were used as untreated controls without implantation of tested PHB/CHIT scaffold (Table 1). The optimal congruence of the defect was achieved by the manual excision of the protruding edges of the implant. After the lavage of the knee with normal saline, the wound was closed in anatomical layers. The described artificial defects and subsequent implantation of biopolymers were performed on four experimental animals. Macroscopic and histologic evaluations of trochlear defects were performed after the procedure.

### 2.4. Monitoring Phase

Standard monitoring was performed after the surgical procedures. Non-steroidal anti-inflammatory drug Flunixin meglumine was administered once a day at a dose of 2.2 mg/kg i.m. (Flunixin a.u.v., Norbrook, Newry, UK) for pain relief. Systemic broad-spectrum antibiotic oxytetracyclinum dihydricum was administered every second day at 20 mg/kg i.m. (Alamycin LA a.u.v., Norbrook, Newry, UK) for infection prophylaxis to the 7 days after the surgery. Wound management included daily local applications of chlorhexidine. All the animals were kept and observed for 6 months after the implantation. 

### 2.5. X-ray Examination

Each experimental trochlear defect was examined by X-ray immediately after the surgery to evaluate the discontinuity of the calcified layer of the cartilage. Further X-ray examinations were performed at intervals of 3 and 6 months after the implantation to evaluate the course of the healing process. Cartilage tissue above the calcified zone is not visible in standard X-ray examinations and it was therefore not possible to evaluate the quality of cartilage healing using this method. The knees of the experimental animals were examined in medio-lateral projection with a Gierth 400 mobile X-ray device (Riesa, Germany) with a focal distance of FAA 100 cm, a voltage of 65–70 kW and a radiation power of 8–10 mAs. The first X-ray examination was performed immediately after the surgery while the experimental animal was still under anesthesia. The subsequent X-rays were performed with manual fixation of the examined limb in a lateral decubitus position with slightly flexed knee.

### 2.6. CT Examination

In four treated sheep a 3D Computer Tomography (CT) scan of the operated knee was performed six months after the implantation (postmortem) to compare the quality of the newly formed tissue with the healthy intact cartilage. Each specific type of tissue has its own specific density. CT scan with contrast is more useful as it offers a better visualization of the cartilage on a live experimental animal. However, in this study it was not possible due to the lack of CT scan parameters for such a measurement. Therefore, cadaveric specimens were examined by non-contrast CT technique. A Metrotom 1500 CT scanner (Carl Zeiss, Oberkochen, Germany) was used with an estimated resolution of 70 µm. Volume Graphics VG Studio MAX 2.2 software was used to evaluate the visual scale of the examined cartilage.

Based on the CT analyses, we evaluated the different density in the areas of the created and subsequently repaired cartilage defects. In contrast to medical tomography, industrial CT uses grey values, not Hounsfield units (HU), for data visualization. The image of the Metrotom 1500 is defined by 2^16^ (0‒65535) grey values. For this reason, relative cartilage density values at selected regions of interest (ROI) were used to analyze the data.

**Figure 3:**
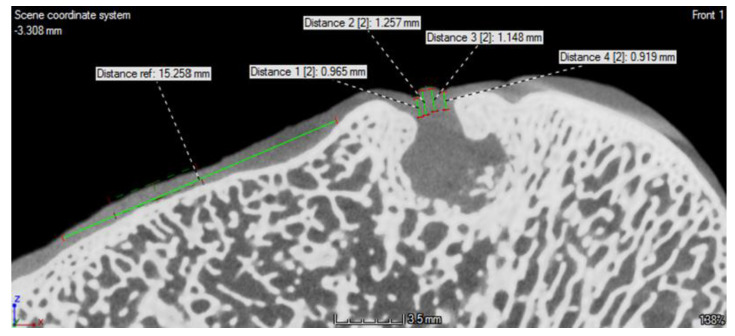
Cartilage density measurement in the defect of the femoral trochlea. Region of interest— Distance 1–4; Reference zone—Distance ref.

**Figure 4:**
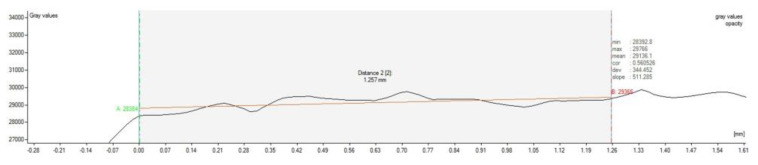
Values of cartilage density at defined profile in measurement zone 2 (Distance 2).

At the ROI, we created 4–5 profile lines along which the density was measured. The number of the lines depended on the width of the defect. The profile line for reference density determination was created in the undamaged tissue in the same slice. To achieve the most precise results, the reference line had the maximum possible length (Figure 3 and Figure 4).

### 2.7. MRI (Magnetic Resonance Imaging) Examination

One of the specimens, which was found to have healed most effectively based on the macroscopic evaluation, was examined six months after the implantation (postmortem). MRI is the most precise imaging method for the detection of chondral defects and the method allows different modalities to be used in the evaluation of the final image. MRI scans were performed on a 1.5 Tesla imaging device (Signa, General Electric Healthcare, Signa, General Electric Healthcare, Illinois, Bloomington, IL, USA) in PD, STIR (short TI inversion recovery), and T2 modes. 

### 2.8. Histology 

Histological examination was performed from the healed defects and from the original intact cartilage in the femoral trochlea. Osteochondral blocks were harvested using a 6 mm cutting tube set of the single-use osteochondral autograft transplant system (OATS) for facilitation of harvesting of various-diameter osteochondral/hyaline cartilage cylinders (Arthrex, Naples, FL, USA) and the formaldehyde fixation of specimens was then performed (10% neutral formaldehyde for one day). After rinsing with water, the samples were decalcified in a 25% chelaton solution (Chelaton III p.a., Centralchem, Bratislava, Slovakia) for one month and dehydrated in ethanol series, cleaned in xylene and embedded in paraffin. The specimens were serially cut at 7 µm using a microtome (Leica RM2244). The Pineda and O’Driscoll histological scoring systems were used to evaluate the quality of the repaired tissue in the cartilage defect. The morphology and architecture of different layers, which are typical for cartilage tissue, were evaluated using hematoxylin-eosin staining, and the presence of glycosaminoglycans in the extracellular matrix was determined in samples using Alcian Blue and Safranine-O staining techniques. The presence of collagen fibers in the samples is detected using Picrosirius Red staining.

#### 2.8.1. Hematoxylin-Eosin Staining

Deparaffinized and hydrated samples were stained in Mayer´s hemalum solution for 20 min (Millipore Sigma, St. Louis, MO, USA). They were then rinsed and stained with 1% eosin solution for 5 min, immediately rinsed again with water and then dehydrated in ethanol series. They were then cleaned in xylene and mounted on slides in a permanent medium (Entellan, Millipore Sigma, St. Louis, MO, USA). 

#### 2.8.2. Alcian Blue Staining

Deparaffinized and hydrated samples were exposed to a 3% acetic acid solution for 3 min, rinsed with water and then stained with an Alcian Blue solution (Millipore Sigma, MO, USA, 1% solution in 3% acetic acid, pH 2.5). Nuclear Fast Red dye (Millipore Sigma, MO, USA) was used to stain the cell nuclei of chondrocytes. The samples were immediately rinsed in water, dehydrated in ethanol series, cleared, and mounted in a permanent medium (Entellan, Millipore Sigma, MO, USA).

#### 2.8.3. Safranin-O Staining

Deparaffinized samples were exposed to Weigert´s hematoxylin for 10 min (Millipore Sigma, St. Louis, MO, USA), rinsed with water for 10 min and stained with Fast Green for 5 min (Millipore Sigma, St. Louis, MO, USA). After a rapid rinsing with a 1% solution of acetic acid, the samples were exposed to Safranin-O dye for 5 min (Millipore Sigma, St. Louis, MO, USA). The samples were then dehydrated, cleaned, and mounted in a permanent medium.

#### 2.8.4. Picrosirius Red Staining

This method is unfortunately unable to detect specific types of collagen fibers. Deparaffinized and hydrated specimens were stained with Weigert’s hematoxylin for 8 min. (Millipore Sigma, St. Louis, MO, USA), rinsed with water, and stained with Sirius Red dye at a concentration of 25% for 1 h (Millipore Sigma, MO, USA). The specimens were then immediately rinsed with an acetic acid solution, dehydrated in 100% alcohol, cleaned, and mounted in a permanent medium (Entellan, Millipore Sigma, St. Louis, MO, USA).

All stained samples were evaluated using a light microscope Olympus CX43 and digital camera 300MIPromicam 3-3CP (Promicra, Prague, Czech Republic).

#### 2.8.5. Histological Scoring Systems

##### Pineda Scoring System

Four objective parameters were evaluated according to the Pineda scoring system: (1) the fulfilling of the defect: 125% (1 point), 100% (0 points), 75% (1 point), 50% (2 points), 25% (3 points), 0% (4 points); (2) the reconstruction of the osteochondral interface: yes (0 points), almost (1 point), not close (2 points); (3) the staining of the extracellular matrix: normal (0 points), reduced staining (1 point), significantly reduced staining (2 points), faint staining (3 points), no stain (4 points); and (4) the cell morphology: normal (0 points), most hyaline and fibrocartilage (1 point), mostly fibrocartilage (2 points), some fibrocartilage, but mostly nonchondrocytic cells (3 points), nonchondrocytic cells only (4 points). The highest possible score is 14 (indicating no healing process), while 0 score indicates complete healing [29]. Ten slides from each repaired defect with the scaffold were evaluated and compared with the control samples from the original non-damaged cartilage harvested from the opposite side of the implanted trochlear groove. The defect, which was healed without biomaterial implantation, was also evaluated and compared with the filled cartilage defect.

##### O’Driscoll Scoring System

The O’Driscoll scoring system was also used to provide more inclusive parameters for the objective evaluation of the cartilage healing quality than the Pineda system: (1) cell morphology: hyaline articular cartilage (4 points), incompletely differentiated mesenchyme (2 points), fibrous tissue or bone (0 points); (2) safranin extracellular matrix staining: normal or nearly normal (3 points), moderate (2 points), slight (1 point), none (0 points); (3) surface regularity: smooth and intact (3 points), superficial horizontal lamination (2 points), fissures 25 to 100% of the thickness (1 point), severe disruption, including fibrillation (0 point); (4) the structure integrity of the newly generated cartilage tissue: normal (2 points), slight disruption including cysts (1 point), severe disintegration (0 point); (5) the linkages between newly generated and original cartilage: bonded at both ends of graft (2 points), bonded at one end or partially at both ends (1 point), not bonded (0 points); (6) hypocellularity: normal cellularity (3 points), slight hypocellularity (2 points), moderate hypocellularity (1 point), severe hypocellularity (0 points); (7) the clustering of chondrocytes: no clusters (2 points), up to 25% of the cells (1 point), 25–100% of the cells (0 points); (8) the cartilage thickness: 100% of normal adjacent cartilage (2 points), 50–100% of normal cartilage (1 point), 0–50% of normal cartilage (0 points); and (9) the presence of degenerative signs in the neighboring tissue: normal cellularity, no clusters, normal staining (3 points), normal cellularity, mild clusters, moderate staining (2 points), moderate cellularity, mild clusters, moderate staining (1 point), severe hypocellularity, poor or no staining (0 points). The final score of the O’Driscoll scoring system is in a range between 0 (no healing) and 24 (complete healing) [29]. In this scoring system, 10 slides from each experimental defect with the scaffold were evaluated and compared with the control samples from the original non-damaged cartilage harvested from the opposite side of the implanted trochlear groove. The defect which was healed without biomaterial implantation (untreated control animals) was also evaluated and compared with the filled cartilage defect. 

##### Polarized Microscopy

Picro Sirius Red staining reacts with collagen by its sulfonic acid groups and basic groups present in the collagen molecule. This connection results in an enhanced birefringency [33]. In addition to photography in transmitted light, pictures of Picrosirius Red staining were taken using 10× and 20× objective using polarized light on an Olympus BX51 upright microscope fitted with DP71 CCD camera. To distinguish coarse mature (red birefringence) and fine immature (green birefringence) collagen fibers, the images were taken individually with different exposure time using red (TRITC) and green (FITC) epifluorescence filters. The images were then adjusted (levels) and assembled in Adobe Photoshop.

### 2.9. Statistical Analyses

GraphPad Prism 6.0 (non-parametric Kruskal–Wallis and Wilcoxon signed-rank test) software was used to perform all the statistical analyses. Values of *p* < 0.05 were considered statistically significant.

## 3. Results

### 3.1. Macroscopic Evaluation of Repaired Cartilage Defects

**Figure 5:**
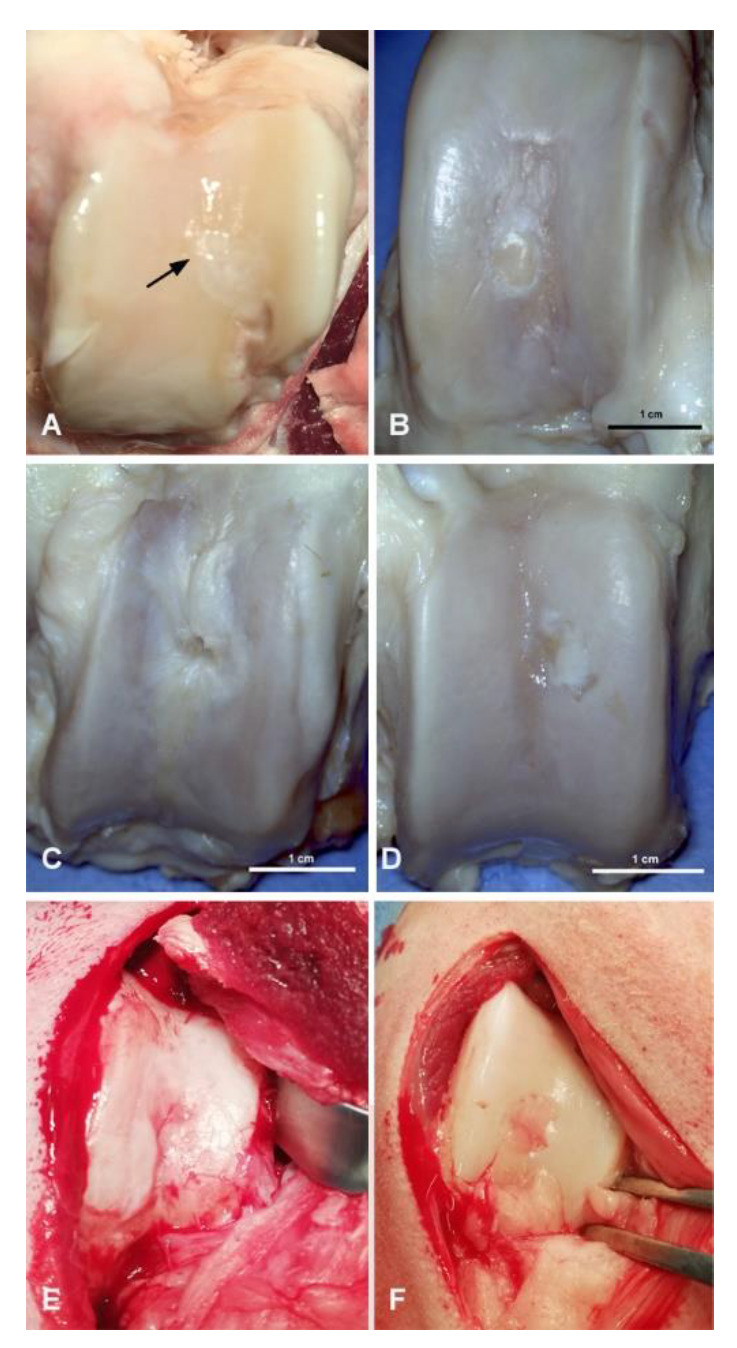
Macroscopic view of the repaired tissue 6 months after the surgery; (**A**–**D**)—The cartilage defects in femoral trochlea filled with PHB/CHIT biomaterial; (**E**,**F**)—The cartilage defects with spontaneous healing without scaffold implantation.

Experimental sheep 1—osteochondral repair defect in the femoral trochlea corresponded well with the level of the surrounding intact cartilage; it was lighter in color, and the consistency of the newly formed tissue was comparable with the original cartilage. The interface site between the regenerated and original tissue was smooth and not easily discerned (Figure 5a).

Experimental sheep 2—osteochondral repair defect had a surface slightly elevated in comparison with the surrounding original cartilage. It was ulcerated with irregular and arthritic-like appearance, probably because of the discongruence of the surface in the newly formed tissue. The color and consistency were comparable with the intact cartilage, while the interface site between the regenerated tissue and original cartilage was clearly visible. (Figure 5b).

Experimental sheep 3—incompletely healed osteochondral defect had a surface more indented in comparison with the intact surrounding tissue. Arthritic-like changes were present again, and the subchondral plate was visible in the center of the defect. The cartilage defect had irregular color, and the interface site between the newly formed and original cartilage was almost invisible. The consistency was comparable with the original cartilage (Figure 5c).

Experimental sheep 4—osteochondral repair defect had a surface corresponding to the intact cartilage tissue, with good congruence and no arthritic-like changes. However, the color of the repaired tissue was lighter and the interface site between the intact and newly formed tissue was clearly visible. The consistency of the repaired tissue was harder than the original cartilage tissue (Figure 5d).

Experimental sheep 5 (untreated control, without implanted scaffold)—spontaneously healed osteochondral defect in the femoral trochlea had a highly irregular surface and clearly visible interface site between the healed defect and the original tissue. The consistency was softer compared with the original cartilage, and had a different slightly red color. Minimal arthritic-like changes were present (Figure 5e).

Experimental sheep 6 (untreated control, without implanted scaffold)—spontaneously healed osteochondral defect had a highly irregular surface indented below the level of the surrounding intact cartilage. The color was also different, appearing slightly reddish. The consistency was softer, and the intervention site was clearly visible (Figure 5f). 

### 3.2. X-ray Evaluation

**Figure 6:**
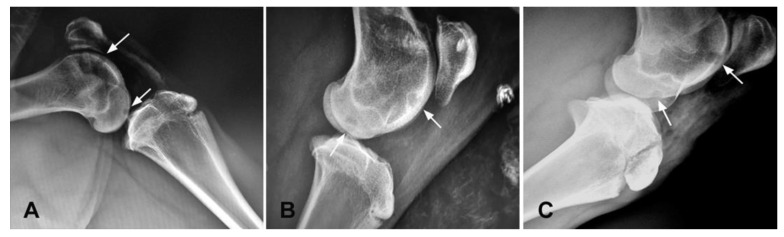
X-ray imaging of the osteochondral defects in femoral trochlea and medial condyle region with implanted, non-contrasting PHB/CHIT scaffold; (**A**)—X-ray image of the osteochondral defects immediately after the surgery and implantation; (**B**)—Osteochondral repair defect six months after the implantation; (**C**)—Osteochondral repair defect without PHB/CHIT scaffold six months after the surgery; arrows—position of osteochondral defects.

Normal bone density and epiphyseal plates were seen in all animals. The depth of the cartilage defect exceeded the calcified subchondral layer. The X-ray examination revealed that the implanted biomaterial remained non-contrasting throughout the whole observation period. The reformation of the calcified layer contour and the regeneration of the bone were clearly visible 6 months after the surgery and implantation. In the X-ray appearance there was no difference between the implanted and non-implanted defects (spontaneous healing) recorded 6 months after the surgery (Figure 6).

### 3.3. CT Evaluation

**Figure 7:**
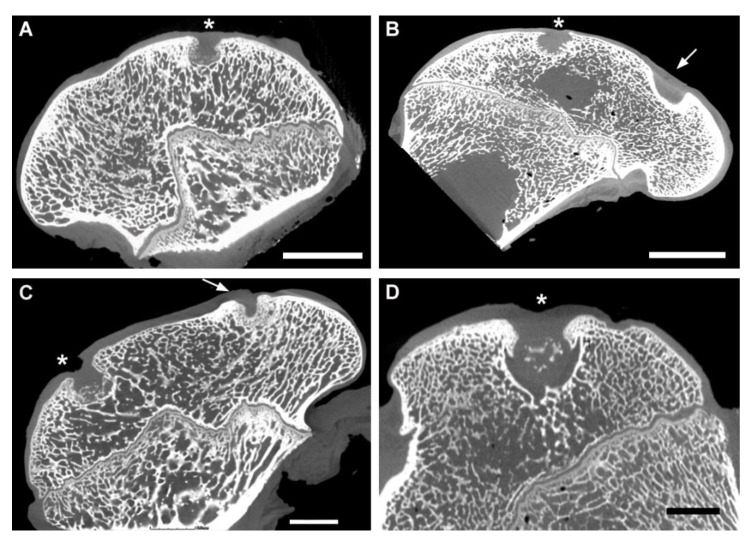
Healing process of the articular cartilage defect 6 month after the PHB/CHIT scaffold implantation. (**A**)—Experimental sheep 1, scale bar: 6.5 mm; (**B**)—Experimental sheep 2, scale bar: 9 mm; (**C**)—Experimental sheep 3, scale bar: 6.5 mm; (**D**)—Experimental sheep 4, scale bar: 6 mm; CT scan; asterisk—site of the defect in the femoral trochlea; arrow—site of the defect in the femoral condyle.

**Figure 8:**
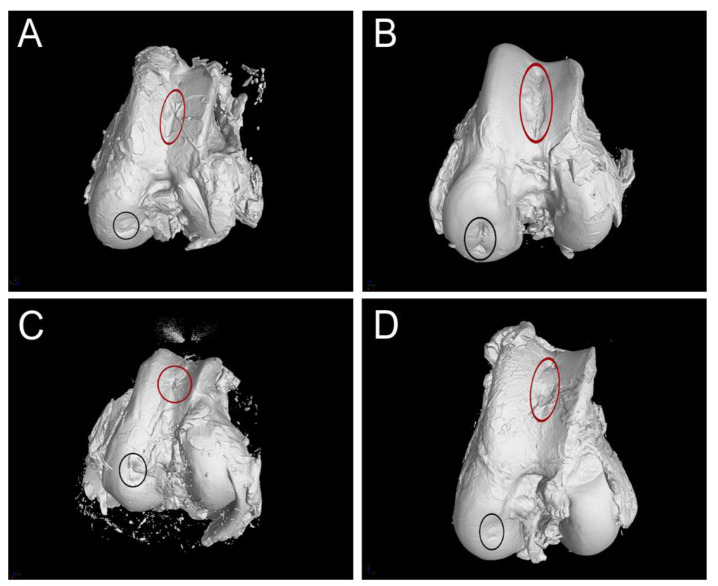
3D visualization of the cartilage repair tissue 6 months after the PHB/CHIT scaffold implantation. (**A**)—Experimental sheep 1; (**B**)—Experimental sheep 2; (**C**)—Experimental sheep 3; (**D**)—Experimental sheep 4; red indication—site of the defect in the femoral trochlea which was studied; black indication—site of the defect in the femoral condyle.

Six months after the surgery, the CT scan images, shown in Figure 7 and Figure 8, demonstrate that the bone defect is not fully regenerated compared to the cartilage layer. The defect is filled with a greater amount of cartilage-like tissue. Some signs of ossification are also present at the base of the defect.

Figure 3 represents cartilage density at examined profile line, region of interest is positioned between green and red vertical line. The red line in the area is created by the least-squares method and defined by the correlation coefficient and slope.

From the values of density for each profile line in analysis of the sheep sample, the average value of density (Average T) was calculated. 

Table 2 shows the average density values for sample ROI (Average T), the average values for the reference profile line, and their percentage difference. Percentage difference is calculated by equation:

(1) $$\mathrm{Percentage}\;\mathrm{difference}=\frac{\mathrm{Average}\;T-\mathrm{Reference}\;\mathrm{average}}{\mathrm{Reference}\;\mathrm{average}}\cdot 100\;\left(\%\right)$$

The difference between total average density for sample and the average reference density was found to be 0.06–4.64% (Table 3).

### 3.4. MRI Evaluation

**Figure 9:**
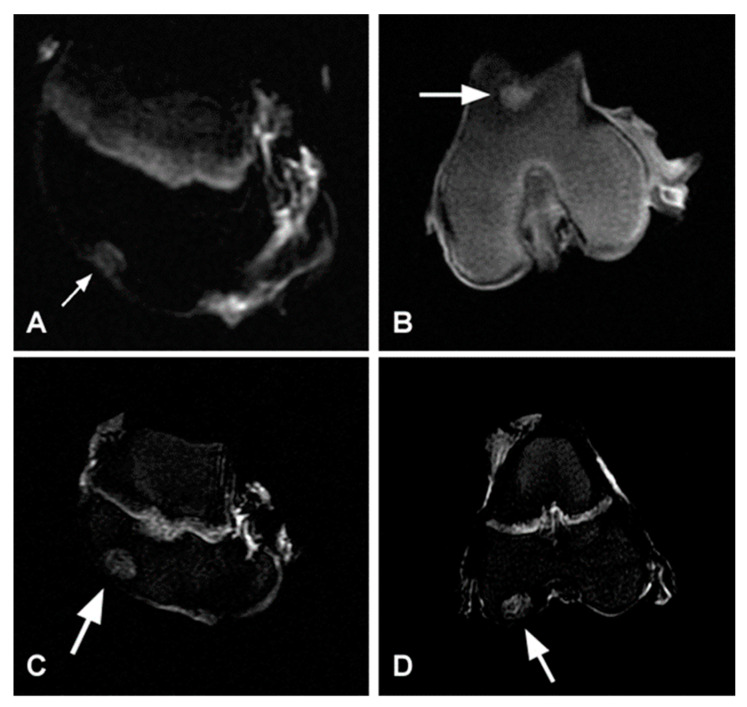
Repaired osteochondral defects 6 months after the PHB/CHIT scaffold implantation (**A**,**B**) and surgery without implantation (**C**,**D**). A—STIR mode, medial condyle– sagittal line; B—T2 mode, femoral trochlea–frontal line; C—Sag STIR mode, medial condyle; D—Cor STIR mode, medial condyle.

Hypersignal tissue was observed in MRI scans of the bone area in the cartilage repair defect with a decrease in the signal recorded towards the superficial zones. In the weight-bearing area of the femoral trochlea, the healing process of the osteochondral defect was observed after scaffold implantation. It showed an almost intact congruency with the articular surface. The signal value of the repaired cartilage tissue was compared with the original cartilage, while the signal of the bone area was also similar to that of the fibrocartilage tissue. A well-repaired osteochondral defect was also present in the femoral condyle. The signal of the bone layer in the defect was similar to the epiphyseal growth plate. The superficial layer was almost identical to that of the intact cartilage. Hypersignal tissue of the untreated cartilage defect was observed with a more visible borderline. The congruency of the surface is interrupted compared to PHB/CHIT-treated defect. (Figure 9).

### 3.5. Histological Evaluation of Repaired Cartilage Defects

**Figure 10:**
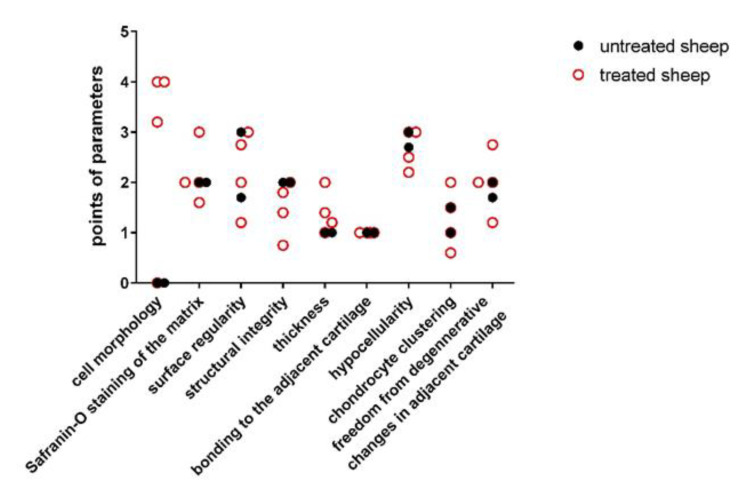
O’Driscoll histological parameters evaluated 6 months after the surgery. Black filled circles—spontaneous repaired defect (experimental sheep 5 and 6); red unfilled rings—defect repair after the implantation of PHB/CHIT scaffold (experimental sheep 1–4).

The most completed regeneration of the osteochondral defect was observed in experimental animals 2 and 3. In the defect of experimental animal 1, the presence of fibrocartilage-like tissue revealed the reduced quality of the healing process and some horizontal lamination (fissures), hypocellular zones, cluster zones of chondrocytes, and irregular width of the cartilage layer. Similarly, reduced quality of the healing process was observed in the case of experimental animal 4. The histological structure of the cartilage tissue was not uniform, and clusters of chondrocytes and acellular areas were present in the superficial zone. The number of glycosaminoglycans (GAGs) in deeper zones was significantly lower and the structure of the matrix was non-homogenous. Several cracks and fibrous tissue were also found in the newly formed tissue. We observed similar signs of tissue structure in the spontaneously healed osteochondral defects without scaffold implantation. The most frequently observed features were the different staining of the matrix, structural irregularities, hypocellularity, clustering of chondrocytes, and degenerative changes in the narrow original cartilage (Table 3, Figure 10).

**Figure 11:**
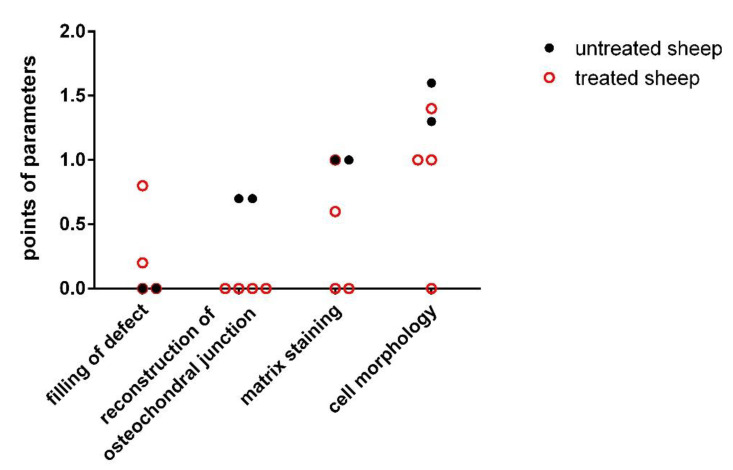
Pineda histological parameters evaluated 6 months after the surgery. Black filled circles—spontaneous repaired defect (experimental sheep 5 and 6); red unfilled rings—defect repair after the implantation of PHB/CHIT scaffold (experimental sheep 1–4).

The higher Pineda score was recorded for the spontaneously healed defects, indicating a poorer quality of the healing. The most frequently observed alteration was the insufficient staining of extracellular matrix and cell morphology, indicating the mixed presence of hyaline and fibrous cartilage (Table 4; Figure 11). On the other hand, excellent quality of repair cartilage tissue was observed in experimental sheep 2 and 3, whereas the quality in experimental sheep 1 and 4 remained rather moderate.

**Figure 12:**
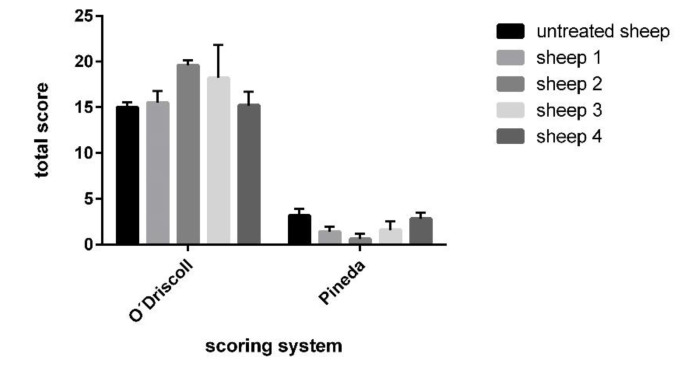
Comparison of results acquired through the histological scoring systems–O’Driscoll and Pineda; untreated sheep mean average score of untreated experimental animals 5 and 6.

When the results of these two scoring systems were compared, both methods provided similar findings, with only distinct differences in the scoring. Experimental sheep 2 and 3 displayed excellent capability for cartilage regeneration in comparison to lower regenerative capacity in experimental animals 1 and 4. The experimental sheep 1 showed a level of the healing process similar to that observed in the untreated experimental animals with spontaneous healing cartilage defects. The untreated healing defects were evaluated using both scoring systems, and they achieved generally poor quality of healing process (Figure 12).

**Figure 13:**
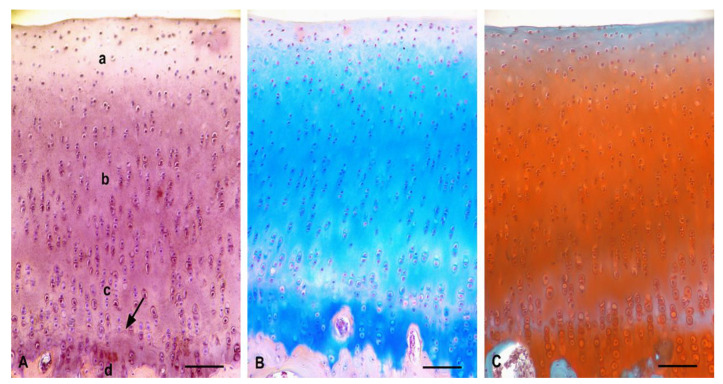
Typical arrangement of intact hyaline cartilage layers. (**A**)—Standard zones of hyaline cartilage, scale bar: 100 µm, staining H–E; (**B**)—Evidence of GAGs presence, scale bar: 100 µm, Alcian Blue staining; (**C**)—Evidence of GAGs, scale bar: 100 µm, Safranin-O staining; a—superficial zone; b—middle zone; c—deep zone; d—calcified zone; arrow—tidemark.

The normal structure of intact articular cartilage with all zones was clearly visible. The superficial zone displayed typical flattened chondrocytes arranged parallel to the articular surface. This zone had a low concentration of GAGs. The middle zone contained randomly spaced spherical chondrocytes, and a higher GAGs content than the superficial zone. The deep zone was characterized by the highest content of GAGs, and the cells were arranged in columns perpendicular to the surface. The tidemark was irregular, and the calcified zone was characterized by a small number of rounded, hypertrophic chondrocytes embedded in a calcified matrix (Figure 13).

**Figure 14:**
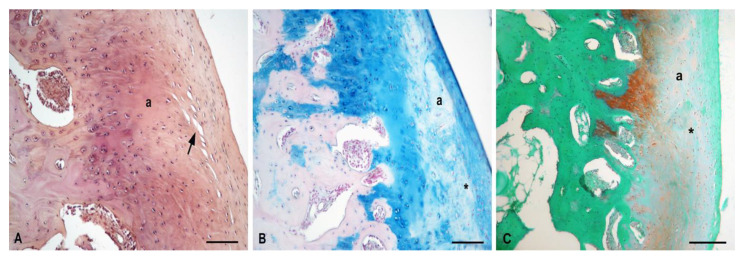
Untreated control animals with spontaneous repair cartilage defect. (**A**)—Staining H–E, scale bar: 200 µm; (**B**)—Evidence of GAGs, Alcian Blue, staining scale bar: 200 µm; (**C**)—Evidence of GAGs, Safranin-O staining, scale bar: 200 µm; a—acellular areas; *—lower intensity of matrix staining; arrow—horizontal fissures.

In untreated sheep 5 and 6 with the spontaneous healing process, the newly formed tissue was not uniform. Defects were partially filled with both fibrous tissue and cartilage-like tissue. The surface of the new tissue was smooth. The superficial layer was formed by fibrous tissue with parallelly arranged cells. In the deeper region, the cartilage-like tissue was formed by hypocellular and acellular areas of different sizes. The deeper zone contained chondrocytes arranged in clusters surrounded with the matrix of a lower-intensity Safranin-O and Alcian Blue staining. Between fibrous and cartilage-like tissue, horizontal fissures were detected. The subchondral layer was intact, comparable to the control group (Figure 14).

#### 3.5.1. Experimental Sheep 1

**Figure 15:**
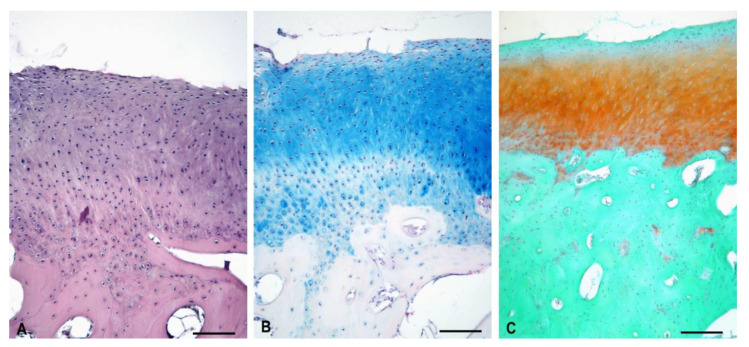
Repair cartilage defect 6 months after the defect after the biomaterial degradation (experimental sheep 1). (**A**)—Cartilage defect repair, H–E staining, scale bar: 200 µm; (**B**)—Evidence of GAGs, Alcian Blue staining with slightly irregular surface; scale bar: 200 µm; (**C**)—Evidence of GAGs, Safranin-O staining with a more fibrous character of the tissue, scale bar: 200 µm.

The cartilage defect was filled with a fibrous-like tissue that contained horizontal or vertical fissures. The surface of the tissue was smooth but slightly irregular. The superficial zone was formed by spindle-shaped cells in an arrangement parallel to the surface, while the deeper zones displayed more oval-shaped cells with chondrocyte morphology. In the middle zone, the matrix was intensely stained with Alcian Blue. In the deep zone the chondrocytes were not arranged into columns. They were organized in lacunae surrounded by extracellular matrix. The structure of the calcified zone and subchondral plate was unchanged (Figure 15). In the middle part of the newly formed tissue, a deep groove extending into the subchondral layer was observed. This groove was surrounded by the fibrous-like tissue, which was hypocellular with a lower GAGs content. The cells in this area were either fibrocytes or chondrocytes. The fibrous tissue extended deeper into the subchondral layer, connecting the fibrocartilage with the bone.

#### 3.5.2. Experimental Sheep 2

**Figure 16:**
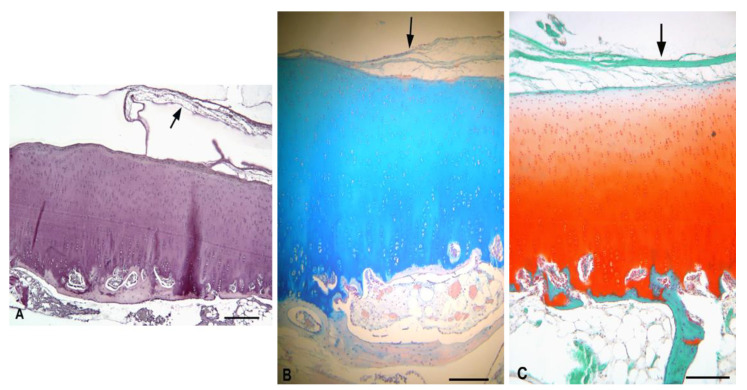
Repair tissue at the site of the cartilage defect after the biomaterial degradation (experimental sheep 2). (**A**)—Cartilage defect filled with the hyaline cartilage H–E staining; (**B**)—Evidence of GAGs presence using Alcian Blue staining; (**C**)—Evidence of GAGs presence using Safranin-O staining; arrow—synovial membrane; *—lower intensity of matrix staining.

The cartilage defect was filled with the hyaline cartilage, which was structurally similar to the intact cartilage. The surface was smooth and covered with a membrane, which was formed by thin parallelly arranged layers. The calcified zone was indistinct, and the subchondral plate was thinner than in the control (Figure 16).

#### 3.5.3. Experimental Sheep 3

**Figure 17:**
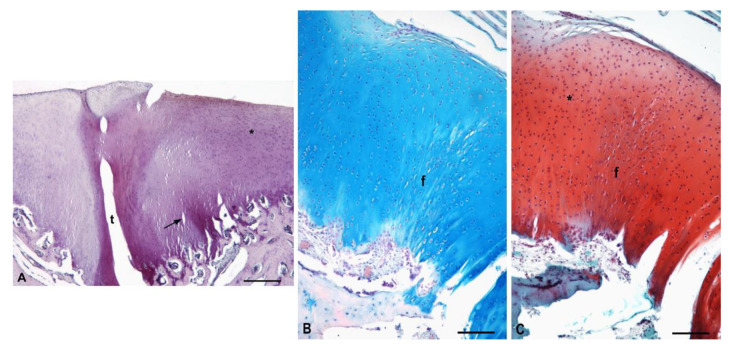
Repair tissue at the site of the cartilage defect after the biomaterial degradation (experimental sheep 3). (**A**)—substantial vertical groove in the middle part, H–E staining, scale bar: 200 µm; (**B**)—Evidence of GAGs, Alcian Blue staining, scale bar: 200 µm; (**C**)—Evidence of GAGs, Safranin-O staining, scale bar: 200 µm; t—vertical rim; f—fibrous-like tissue; arrow—small fissures; *—hypercellularity.

The cartilage defect was irregularly filled with the cartilage-like tissue. It was stained intensely with both AB and Safranin-O, indicative of a higher number of GAGs. In the middle part of the newly formed tissue, a vertical groove was observed. The groove extended through all zones except of the superficial level, which remained almost intact. Fibrous-like tissue was detected at the site of the groove. Towards the periphery of the defect, the new tissue was rather fibrous with hypercellular areas. The subchondral plate was thinner than in the intact cartilage (Figure 17).

#### 3.5.4. Experimental Sheep 4

**Figure 18:**
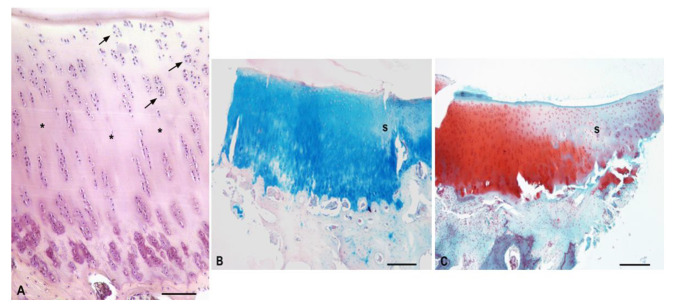
Repair tissue at the site of the cartilage defect after the biomaterial degradation (experimental animal 4). (**A**)—Clusters of chondrocytes and acellular areas in the newly formed tissue, H–E staining, scale bar: 100 µm; (**B**)—Evidence of GAGs, Alcian Blue staining, scale bar: 500 µm; (**C**)—Evidence of GAGs, Safranin-O staining, scale bar: 500 µm; z—lower staining of the matrix; arrow—clusters of chondrocytes; *—acellular areas.

The lowest level of regenerative capacity was observed in experimental animal 4. The histological structure of the regenerating hyaline-like tissue was not uniform, yet the surface of the new tissue was smooth. Formation of individual zones was indistinct, and the superficial layer contained clusters of chondrocytes, which were alternating with acellular areas. This is probably the result of insufficient time period for correct and typical organization of cartilage structure. These changes became more pronounced towards the periphery of the defect, where the new tissue stained less intensely for GAGs. Several grooves were observed in the middle and deep zones, some of which extended to the calcified layer. Profound structural changes were observed in subchondral region, where the trabecular bone was substituted by connective tissue (Figure 18).

#### 3.5.5. Polarized Microscopy Evaluation of Collagen

**Figure 19:**
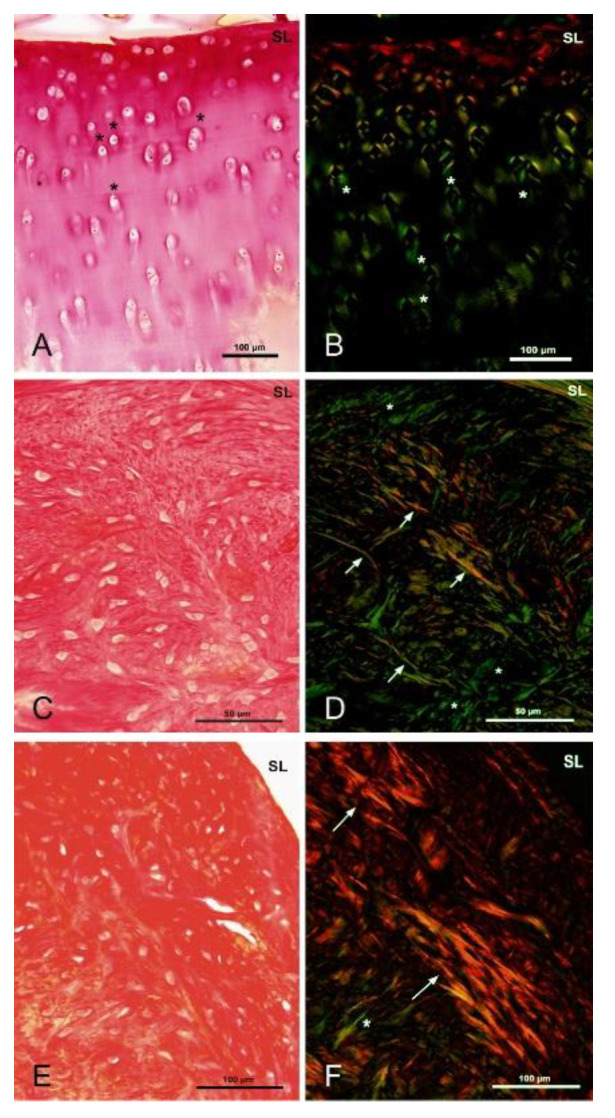
Monitoring of collagen fibers. A, B—Healthy sheep articular cartilage: production of new collagen in the area surrounding the chondrocytes in the intermediate layer of the articular cartilage (green), surface layer with mature collagen fibers running parallel to the joint surface (red), staining of collagen with Picrosirius Red in transmitted (**A**) and polarized (**B**) light, scale bar: 100 µm; asterisk (**A**)—chondrocytes, asterisk (**B**)—newly formed collagen; (**C**,**D**)—Experimental sheep articular cartilage with biomaterial: weaker production of collagen around the chondrocytes in the intermediate layer of the articular cartilage (green), presence of thick mature bundles of collagen fibers arranged parallel to each other in several layers (red)—superficial and intermediate layers, staining of collagen with Picrosirius Red, scale bar: 50 µm; *—new formed collagen, arrows—collagen fibers; (**E**,**F**)—Experimental sheep articular cartilage with spontaneous healing without biomaterial: formation of fibrocartilage with thick bundles of collagen fibers arranged parallel to each other in several layers (red)—superficial and intermediate layers, staining of collagen with Picrosirius Red, scale bar: 100 µm; *—new formed collagen; arrows—collagen fibers, SL—superficial layer of cartilage.

Newly formed collagen fibers in the normal articular cartilage were situated around in the chondrocyte territorial matrix of the chondrocyte intermediate layer. The superficial layer of the healthy (intact) cartilage consisted of mature collagen fibers running parallel to the joint surface (Figure 19A,B). The lowest amount of new collagen fibers was observed in the unfilled osteochondral defect. Furthermore, the thickest collagen fibers and decreased number of chondrocytes were observed in the unfilled cartilage defect as a sign of fibrocartilage (Figure 19E,F). 

Presence and formation of the collagen fibers were also reduced in the chondral defects filled with PHB/CHIT material. After the implantation of material, newly repaired tissue consisted of chondrocytes that were aligned with the thick collagen fibers. Formation of new collagen fibers was reduced compared to intact cartilage (Figure 19C,D). 

## 4. Discussion

Our in vivo study presents the first evidence of biological integration-implanted PHB/CHIT-based acellular porous scaffold into an artificial osteochondral defect in femoral trochlea. This study presents detailed analysis of articular cartilage repair process with chitosan-based scaffold using non-invasive imaging techniques (X-ray, CT, MRI) in combination with the histological evaluation. Using both techniques, we observed completed degradation of PHB/CHIT biomaterial. Please note that in vitro testing of FTIR analysis (Fourier Transform Infrared Spectroscopy) and enzymatic degradation of PHB/chitosan blends [30,31] clearly showed the formation of a large fraction of fine amorphous PHB as well as the rapid degradation of chitosan with lysozyme which is normally present in human body fluids. Because the prepared blends represent weak chemically bonded biopolymer matrix, they can be characterized in a good approximation as physically bonded precipitates that are debonded after the degradation of one of the biopolymers. This fact probably significantly affected the biodegradation of used blends after implantation. Hyaline-like cartilage tissue was observed in most scaffold-treated experimental animals. However, one implanted defect was repaired with the fibrocartilage, compared with the spontaneously healed untreated defect. 

Microscopy is the most accurate method for evaluating cartilage damage and regeneration. The evaluation of the histologically stained sections provided a method to determine how closely the morphology and organization of the repaired cartilaginous tissue resemble physiological articular cartilage. Using more than one scoring system provides more information about the histological characteristics of the regenerated tissue [34]. It was concluded by Moojen et al. (2002) [28] that both O’Driscoll and Pineda scores were reliable semi-quantitative cartilage scoring systems with good correlation. The minor differences observed in the results of these histological scoring systems are likely because the Pineda system is more basic, with only four morphological parameters, while the O’Driscoll system is more precise and measures nine morphologic parameters. It is important to note that scoring systems are generally considered to be a highly subjective method of evaluation. The largely indicative results obtained with this method may not correspond to the accuracy provided by a detailed histological evaluation.

For preclinical and clinical repair of cartilage, histological scoring provides a crucial outcome measure. Our study results, which we obtained using the histological scoring systems of O’Driscoll and Pineda, were confirmed with individual histological evaluation. All samples were devoid of inflammatory infiltration at the time of sampling. In three treated samples, the defect was filled with cartilage-like tissue that showed signs of hyaline cartilage. The surface of the regenerated tissue was generally smooth, but in some areas it was also slightly irregular. In particular, the presence of high-quality hyaline cartilage is clinically important since lower-quality fibrocartilage may degenerate after a few years and does not tolerate mechanical loads over time, which is connected with the risk of developing secondary osteoarthritis [35]. It is also important to note that regenerated hyaline cartilage may still fail if mechanical irritation persists due to malalignment, poor defect filling, or if the surface is disintegrated. Many characteristics are just as vital as the extracellular matrix composition when determining the quality of the newly formed tissue. Important characteristics that are needed for the maintenance of hyaline cartilage are cell organization, presence of viable cells with zone-specific architecture, proper density of cells, surface integrity, cartilage-bone integration, and the orientation and organization of the collagen fibrils [36].

The collagen fibers display differential organization in the different layers of cartilage, running parallel to the articulating surface in the superficial layer, appearing rather disorganized in the middle layer and then running vertically across the depth of the transitional zone [37]. Pericellular matrix of chondrocyte is in a contact with surrounding territorial matrix that contains a weave of small-diameter fibrils oriented parallel to the cell surfaces [38,39]. Our observations using polarized microscopy visualized collagen fibers architecture in the cartilage layers. Newly formed collagen fibers in the intact sheep articular cartilage were situated in chondrocyte territorial matrix of the intermediate layer. In the superficial layer the collagen fibers ran parallel to the surface of cartilage. In the PHB/CHIT-treated cartilage defect, we observed a presence of collagen fibers also in the intermediate layer, suggesting a beginning of fibrocartilage formation.

Several authors studied the effect of chitosan on regeneration of the articular cartilage. Previously, chitosan scaffolds enriched with D−(+) raffinose were evaluated in osteochondral defects conducted in rabbit distal femurs (both on the condyle and on the trochlea) after 2 or 4 weeks [40]. They observed a fibrous capsule surrounding the implants and inflammatory infiltrate. No hyaline cartilage was formed in the defects and the defects were not completely repaired after being filled with chitosan. Similarly, fibrocartilage tissue was observed after the implantation of a hybrid magnesium-doped hydroxyapatite (MgHA), collagen, chitosan-based scaffold, which was tested in a sheep model. The microtomography analysis revealed non-uniform new bone growth with large cystic areas around the residual material [41]. Another study tested freeze-dried (FD) chitosan implants in full-thickness cartilage defects in medial femoral condyles of 8–9-year-old sheep. At 9 months after the implantation, biomaterial supported bone plate repair and stimulated 68% cartilage resurfacing. Both treated and control cartilage repair tissues had lower glycosaminoglycan content than intact cartilage and were thinner, stiffer, and more permeable [42].

Furthermore, implantation of chitosan–agarose–gelatin cryogel scaffold showed significant cartilage regeneration in female New Zealand white rabbits [43]. The PHB/CHIT scaffolds did not elicit any adverse immunological reaction as shown by hematological analysis. In this context, we also evaluated acute phase protein concentrations where we observed decreasing tendency to preoperative values already four weeks after the PHB/CHIT biomaterial implantation; thus, the inflammatory reaction was no longer present [32]. We also demonstrated successful preclinical testing of PHB/CHIT-based scaffold using the chick chorioallantoic membrane assay [44].

There is a high degree of functional and morphological similarity between sheep cartilage and human cartilage in the terms of structure, thickness, and density, and therefore sheep is considered one of the most convenient animals for use in experimental studies of the healing chondral defects with the use of different biomaterials. However, we need to consider several limitations associated with the ovine model. For instance, the articular cartilage thickness in sheep can range between 0.4‒1.6 mm [3,45]. This could lead to variable results within a study since this variability makes the volume of the defect created in the cartilage and subchondral bone likely to be different between individual animals. Furthermore, the cartilage thickness is different regarding implantation site. Medial femoral condyle has the thicker cartilage (1096 µm) compared to cartilage of trochlea (up to 780 µm) [46]. These results correspond with our measures, when average thickness of trochlear intact cartilage was 758 µm. Average cartilage thickness of treated defect was significantly higher (1233 µm) compared with intact cartilage and untreated defect as well (872 µm). In relation to this fact, we found different cartilage density among treated defects, and it is likely due to underlying variable thickness of the cartilage. Smaller animals such as rodents or rabbits are generally accepted and used as a small animal model in initial lines of investigation. Nevertheless, in the final preclinical evaluation of a reconstruction technique for articular cartilage, confirmation in a large animal model is often required. Currently, no perfect preclinical animal model exists, but the authors concluded that the sheep is a readily accessible model for cartilage repair studies despite the above-mentioned limitations [47].

Tissue engineering as a rapidly expanding field provides alternatives for articular cartilage repair and regeneration through developing biomimetic tissue substitutes [48]. The terminal goal of such new cartilage repair approaches is to repair and regenerate osteochondral tissue with the support of the biochemical and biophysical environment of the extracellular matrix in damaged articular cartilage [49]. Research has demonstrated the potential of biomaterial physico-chemical properties significantly influencing the proliferation, differentiation, and matrix deposition by progenitor cells [50].

Biomaterial may act as a template for repair tissue formation resulting in regeneration of hyaline cartilage tissue. Small subchondral drill holes that reflect the physiological trabecular distance improve osteochondral repair in a translational model more effectively than larger drill holes [51]. In our study, we used acellular PHB/CHIT scaffold and subchondral drilling due to the release of the bone marrow stem cells. We also observed that this technique supported formation of the hyaline-like cartilage. Clinical studies showed improved cartilage regeneration when using biomaterials for implantation after bone marrow stimulation in animal models. From this point of view, cartilage regeneration is more effective with implantation of acellular biomaterials in microfracture defects compared to microfracturing alone. The efficacy is further improved by the incorporation of stimulating factors [52]. These findings are in line with our study; thus, ideally, biomaterial should achieve regeneration by stimulating the recruitment of cells from the bone marrow and provide the biochemical and physical clues that direct the cells to regenerate and build up the zones of articular cartilage to exhibit normal morphology [53]. 

Cartilage in sheep is thinner compared to human cartilage and the creation of defects is possible in a smaller area that it would be to be clinically significant in humans. Translational data from animal studies to clinical trials depend on the comparability to the clinical situation, which in our case of 6 months seems to be a short monitoring time. Since the clinical improvements in humans is frequently observed up to one and a half years after surgery [54], the short six-month lasting period may be considered the limitation of our study.

The results suggest that the assessment of the cartilage healing process using a combination of scoring systems can indicate a level of regeneration and quality of the repair tissues. However, histological analysis remains the best method for scoring and describing the properties of the structure and morphological characteristics of the new formed hyaline cartilage.

## 5. Conclusions

Acellular PHB/CHIT porous biomaterial used in this study supported osteochondral regeneration mostly by the formation of hyaline-like cartilage. Only one experimental animal showed a repairing process by fibrocartilage tissue formation. We observed the total degradation of biomaterial in the knee articular cartilage defect with microfractures. The healing process took place without inflammation and significant changes of cartilage density. Half the treated experimental animals achieved satisfactory evaluation using the histological scoring system. Given the limited number of animals involved in this study, PBH/CHIT-based porous biomaterial shows considerable promise in the one-stage surgical procedure of osteochondral lesions of non-degenerative etiology. Nevertheless, our current in vivo data encourage further animal studies designed based on a higher number of animals. In combination with bioactive factors (stem cells, growth factors, cytokines), it could help improve the cartilage tissue regeneration associated with cartilage damage.

## 6. Patents

There is a patent resulting from the work reported in this manuscript: Lubomir Medvecky, Maria Giretova, Eva Petrovova: Biopolymer composite system for cartilage regeneration: Published patent application no. 89-2014. Banska Bystrica: Industrial Property Office of the Slovak Republic, 01.07.2016.

## Figures and Tables

**Table 1:** Scheme of the surgical procedure and implantation of PHB/CHIT biomaterial in using experimental animals.

Animals	Defects Location	Scaffold Implantation
**Experimental sheep 1**	left trochlea	+
	left condyle	+
**Experimental sheep 2**	left trochlea	+
	left condyle	+
**Experimental sheep 3**	left trochlea	+
	left condyle	+
**Experimental sheep 4**	left trochlea	+
	left condyle	+
**Experimental sheep 5**	left trochlea	-
	left condyle	-
**Experimental sheep 6**	left trochlea	-
	left condyle	-

**Table 2:** Values of density along the reference line; REF—reference value.

Sample	Average T	Reference Average	Percentage Difference (%)
**Experimental sheep 1**	28,559.66	28,659.53	0.35
**Experimental sheep 2**	38,320.75	39,369.86	2.67
**Experimental sheep 3**	29,388.88	29,407.09	0.06
**Experimental sheep 4**	34,143.07	35,803.56	4.64

**Table 3:** Evaluation of healing process according to O´Driscoll histological scoring system

Parameters	Untreated Sheep 1	Untreated Sheep 2	Experimental Sheep 1	Experimental Sheep 2	Experimental Sheep 3	Experimental Sheep 4
**Degenerative changes in adjacent cartilage**	1.67 ± 0.58	2.00 ± 0.00	1.20 ± 0.45	2.00 ± 0.00	2.00 ± 0.71	2.75 ± 0.50
**Chondrocyte clustering**	1.00 ± 0.00	1.50 ± 0.58	0.60 ± 0.55	1.00 ± 0.00	2.00 ± 0.00	1.50 ± 0.60
**Hypocellularity**	2.67 ± 0.58	3.00 ± 0.00	2.20 ± 0.45	3.00 ± 0.00	3.00 ± 0.00	2.50 ± 0.60
**Bonding to the adjacent cartilage**	1.00 ± 0.00	1.00 ± 0.00	1.00 ± 0.00	1.00 ± 0.00	1.00 ± 0.00	1.00 ± 0.00
**Thickness of cartilage**	1.00 ± 0.00	1.00 ± 0.00	1.20 ± 0.45	2.00 ± 0.00	1.40 ± 0.55	1.00 ± 0.00
**Structural integrity**	2.00 ± 0.00	2.00 ± 0.00	1.80 ± 0.45	2.00 ± 0.00	1.40 ± 0.89	0.75 ± 0.96
**Surface regularity**	1.67 ± 1.15	3.00 ± 0.00	1.20 ± 0.45	3.00 ± 0.00	2.00 ± 0.70	2.75 ± 0.50
**Safranin-O staining of the matrix**	2.00 ± 0.00	2.00 ± 0.00	2.00 ± 0.00	1.60 ± 0.55	2.00 ± 0.00	3.00 ± 0.00
**Cell morphology**	0.00 ± 0.00	0.00 ± 0.00	4.00 ± 0.00	4.00 ± 0.00	3.20 ± 1.79	0.00 ± 0.00
**Total average score**	**13.00 ± 2.00**	**15.50 ± 0.58**	**15.20 ± 1.48**	**19.60 ± 0.55**	**18.20 ± 3.63**	**15.50 ± 1.29**

**Table 4:** Evaluation of healing process according to Pineda histological scoring system.

Parameters	Untreated Sheep 1	Untreated Sheep 2	Experimental Sheep 1	Experimental Sheep 2	Experimental Sheep 3	Experimental Sheep 4
**Cell morphology**	1.33 ± 0.52	1.80 ± 0.49	1.00 ± 0.00	0.00 ± 0.00	1.00 ± 0.00	1.40 ± 0.55
**Staining of the matrix**	1.17 ± 0.41	1.00 ± 0.00	1.00 ± 0.00	0.6 ± 0.55	0.20 ± 0.45	0.00 ± 0.00
**Reconstitution of the osteochondral junction**	0.83 ± 0.41	0.29 ± 0.49	0.00 ± 0.00	0.00 ± 0.00	0.00 ± 0.00	0.00 ± 0.00
**Filling of the defect**	0.00 ± 0.00	0.00 ± 0.00	0.80 ± 0.84	0.00 ± 0.00	0.40 ± 0.55	0.00 ± 0.00
**Total average score**	**3.33 ± 0.82**	**3.00 ± 0.58**	**2.80 ± 0.75**	**0.60 ± 0.55**	**1.20 ± 0.45**	**1.40 ± 0.55**

## Data Availability

Data presented in this study are available on request from the corresponding author. The data are not publicly available due to the fact that these data are published for the first time and authors have no problems to provide them on request.
